# Inhibitory Effects of *Terminalia catappa* on UVB-Induced Photodamage in Fibroblast Cell Line

**DOI:** 10.1155/2011/904532

**Published:** 2010-10-14

**Authors:** Kuo-Ching Wen, I-Chen Shih, Jhe-Cyuan Hu, Sue-Tsai Liao, Tsung-Wei Su, Hsiu-Mei Chiang

**Affiliations:** Department of Cosmeceutics, China Medical University, Taichung 40402, Taiwan

## Abstract

This study investigated whether *Terminalia catappa* L. hydrophilic extract (TCLW) prevents photoaging in human dermal fibroblasts after exposure to UVB radiation. TCLW exhibited DPPH free radical scavenging activity and protected erythrocytes against AAPH-induced hemolysis. In the gelatin digestion assay, the rates of collagenase inhibition by TCL methanol extract, TCLW, and its hydrolysates were greater than 100% at the concentration of 1 mg/mL. We found that serial dilutions of TCLW (10–500 *μ*g/mL) inhibited collagenase activity in a dose-dependent manner (82.3% to 101.0%). However, TCLW did not significantly inhibit elastase activity. In addition, TCLW inhibited MMP-1 and MMP-9 protein expression at a concentration of 25 *μ*g/mL and inhibited MMP-3 protein expression at a concentration of 50 *μ*g/mL. TCLW also promoted the protein expression of type I procollagen. We also found that TCLW attenuated the expression of MMP-1, -3, and -9 by inhibiting the phosphorylation of ERK, JNK, and p38. These findings suggest that TCLW increases the production of type I procollagen by inhibiting the activity of MMP-1, -3 and -9, and, therefore, has potential use in anti-aging cosmetics.

## 1. Introduction

The leaves of *Terminalia catappa* L. (Combretaceae) are commonly used as folk medicine in Southeast Asia to treat dermatitis and hepatitis [1]. Extracts of the leaves and bark of the plant have been reported to have chemopreventive [2, 3], antioxidant, and superoxide radical scavenger effects [4], anti-HIV reverse transcriptase activity [5], and hepatoprotective [4] and anti-inflammatory effects [6]. *Terminalia catappa *extract has also been shown to decrease the protein expression of MMP-2 and MMP-9 in lung cancer cells [3]. The phytochemicals in this plant include tannins (punicalagin, punicalin, terflavins A and B, chebulagic acid), flavanoids (isovitexin, vitexin, isoorientin, rutin) and triterpenoids (ursolic acid and asiatic acid) [7–9]. The hepatoprotective activity of *Terminalia catappa *extract has been shown to be due primarily to the superoxide anion and hydroxyl radical scavenging activity of ursolic acid and asiatic acid [10].

Wrinkles, laxity, and hyperpigmentation characterize aging [11]. Skin aging can be divided into two basic processes, intrinsic, and extrinsic aging. Extrinsic aging is generally referred to as photoaging due to chronic exposure to short wavelength UV light (UVB) and is characterized by severe wrinkling and pigmentary changes, such as solar lentigo and mottled pigmentation on exposed areas such as the face, neck, and forearm. The most abundant structural protein in skin connective tissue is type I collagen, which is synthesized primarily by fibroblasts and is responsible for conferring strength and resiliency [12]. Ultraviolet- (UV-) induced skin damage principally manifests as degradation of extracellular matrix (ECM) proteins, including type I collagen, elastin, proteoglycans, and fibronectin [13, 14]. It has been shown that UV irradiation leads to the formation of reactive oxygen species (ROS) that activate the mitogen-activated protein (MAP) kinase pathway, which subsequently induces the expression and activation of matrix metalloproteinases (MMPs) in human skin *in vivo* [15, 16]. MMPs are known to be upexpressed in human fibroblasts within hours after exposure to UV irradiation and are, therefore, considered key factors in the photoaging process. Therefore, agents with the ability to elevate ECM protein levels or inhibit the major collagen-degrading enzymes like MMPs would prove to be useful in the development of effective anti-aging agents. 

This study investigated the effects of *Terminalia catappa* extract and its hydrolysates on protein expression of MMPs, elastase, and type I procollagen in human dermal fibroblasts after exposure to UVB and investigated the mechanism by which the extract protects against photodamage.

## 2. Material and Methods

### 2.1. Chemicals

Leaves of *Terminalia catappa *L. were harvested in Taichung County, Taiwan. Human foreskin fibroblasts were obtained from the Bioresource Collection and Research Center (Hsinchu, Taiwan). Bradford Reagent was supplied by Bio-Rad Laboratories (CA, USA). Elastase substrate IV and porcine elastase were purchased from Calbiochem (San Diego, CA, USA). Coomassie blue R-250, dibasic sodium phosphate, Igepal CA-630, tris and 3-(4,5-dimethylthiazol-2-yl)-2,5-diphenyltetrazolium bromide (MTT) were purchased from USB (Cleveland, OH, USA). Collagenase was purchased from Calbiochem, Merck (Darmstadt, Germany). Fetal bovine serum (FBS), penicillin-streptomycin, trypsin-EDTA, and Dulbecco's Modified Eagle's Medium (DMEM) were purchased from Gibco, Invitrogen (Carlsbad, CA, USA). Fluorogenic Peptide Substrate I was purchased from R&D systems (Wiesbaden, Germany). Gelatin, agarose, hydrochloric acid, methanol, dimethyl sulfoxide (DMSO), propylene glycol (PG), doxycycline hyclate, calcium chloride (CaCl_2_), 1,1-diphenyl-2-picrylhydrazy (DPPH) and 2,2′-azobis (2-methylpropionamidine) dihydrochloride (AAPH), and DL-dithiothreitol were purchased from Sigma-Aldrich Chemicals (St. Louis, MO, USA).

### 2.2. Preparation of *Terminalia catappa* L. Extract and Its Hydrolysates

 The dried leaves were ground and then extracted twice with a 30-fold volume of methanol for 1 h by ultrasonication. The supernatant was filtered and then evaporated to dryness in vaucum to obtain methanol extract of *Terminalia catappa *L. (TCLM). The hydrophilic extract of *Terminalia catappa *L. (TCLW) was prepared in the same manner as TCLM except that the solvent was replaced with water. Acid hydrolysis of TCLW was carried out at 80°C in the presence of 1.2 N HCl (2 mL) for 30 min and 2.4 N HCl (2 mL) for 60 min. After hydrolysis, the solution was partitioned with ethyl acetate (EA). The EA layer was evaporated to dryness in vaucum to obtain TCLW hydrolysate (TCLWH). The abbreviations used for and the hydrolytic conditions of TCLWH are as follows: TCLWH1, 0.6 N HCl for 0.5 h; TCLWH2, 1.2 N HCl for 0.5 h; TCLWH3, 0.6 N HCl for 1 h; TCLWH4, 1.2 N HCl for 1 h. The TCLW extract and TCLW hydrolysates were stored at −20°C before use.

### 2.3. Total Phenolic Content of TCLW

Total phenolic content of TCLW was determined by the Folin-Ciocalteu reaction. TCLW was mixed with 2-fold 10% Folin-Ciocalteu phenol reagent. The mixture was allowed to stand at room temperature for 5 min and then sodium carbonate (700 mM) was added to the mixture. The resulting blue complex was then measured at 760 nm. Gallic acid was used as a standard for the calibration curve. The phenolic compound contents were calibrated using the linear equation based on the calibration curve. The contents of phenolic compounds are expressed as *μ*g gallic acid equivalent/mg TCL dry weight.

### 2.4. DPPH Radical Scavenging Activity of TCLW

Reaction mixtures containing 200 *μ*M DPPH (100 *μ*L) and serial dilutions of sample (concentration of sample ranging from 25 to 1000 *μ*g/mL) were placed in a 96-well microplate at room temperature in the dark for 30 min. After incubation, the absorbance was read at 517 nm by an ELISA reader (Tecan, Grodig, Austria). Ascorbic acid was used as the positive control. Scavenging activity was determined by the following equation:

(1) $$\begin{array}{cc} & \text{Scavenging}\;\text{effect}\;\text{\%}\;\left(\text{capacity}\;\text{to}\;\text{scavenging}\;\text{the}\;\text{DPPH}\;\text{radical}\right)\\ & \;\;=\left(\frac{\left(A_{\text{control}\;\text{at}\;517\;\text{nm}}-A_{\text{blank}\;\text{at}\;517\;\text{nm}}\right)-\left(A_{\text{sample}\;\text{at}\;517\;\text{nm}}-A_{\text{blank}\;\text{at}\;517\;\text{nm}}\right)}{\left(A_{\text{control}\;\text{at}\;517\;\text{nm}}-A_{\text{blank}\;\text{at}\;517\;\text{nm}}\right)}\right)\times 100. \end{array}$$

### 2.5. Preparation of Erythrocyte Suspensions and Hemolysis Assay

Whole blood was obtained from male SD rats via cardiopuncture and collected in an EDTA-containing tube. The erythrocytes were isolated by centrifugation at 3000 × g for 10 min, washed four times with PBS, and then resuspended to the desired hematocrit level using the same buffer. In order to induce free radical chain oxidation in the erythrocytes, aqueous peroxyl radicals were generated by thermal decomposition of AAPH in oxygen. An erythrocyte suspension at 5% hematocrit was incubated with PBS (control) or preincubated with TCLW (10–50 *μ*g/mL) at 37°C for 30 min, followed by incubation with or without 25 mM AAPH in PBS at pH 7.4. This reaction mixture was shaken gently while being incubated for a fixed interval at 37°C. A 200-*μ*L aliquot of the reaction mixture was removed and centrifuged at 3000 ×g for 2 min, with absorbance of the supernatant determined at 540 nm. Reference values were determined using the same volume of erythrocytes in a hypotonic buffer (5 mM phosphate buffer at pH 7.4; 100% hemolysis). The hemolysis percentage was calculated using the formula: [(*A*_sample_/*A*_control_)] × 100.

### 2.6. Gelatin Digestion Assay

Agarose solution (1%) was prepared in collagenase buffer (50 mM Tris-HCl, 10 mM CaCl_2_, 0.15 M NaCl, pH 7.8) with 0.15% porcine gelatin (Sigma Aldrich, Cat. G-2500) and allowed to solidify on plates. Various concentrations of TCLW and TCLWH (10 *μ*L) dissolved in 10% DMSO were incubated with 10 *μ*L of bacterial collagenase-1 (0.1 mg/mL) in 80 *μ*L of collagenase buffer for 1 h at room temperature. Doxycycline hyclate was used as positive control. The samples (40 *μ*L) were loaded onto paper disks placed on gelatin-agarose gel and incubated for 18 h at 37°C. The degree of gelatin digestion in agarose gel was visualized by Coomassie Blue staining after removal of the paper disks. Following destaining, the area of the light translucent zone over blue background was determined by a densitometric program (TINA) to estimate gelatinase activity.

### 2.7. MMP Activity Assays

Enzyme activity assays were performed in 50 mM tris buffer (pH 7.8), 0.15 M NaCl, and 10 mM CaCl_2_. Various concentrations of TCLW and TCLWH were tested for their ability to digest a synthetic fluorogenic substrate (a general MMP substrate). Each concentration of TCLW and TCLWH was incubated with 1 *μ*M substrate at 37°C for 20 h. Fluorescence intensity was measured at 320 nm (excitation) and 450 nm (emission) with a fluorescence reader. The rate of collagenase inhibition was calculated by the following equation:

(2) $$\text{Inhibition}\;\left(\text{\%}\right)=\left(1-\frac{\left(\text{C}-\text{D}\right)}{\left(\text{A}-\text{B}\right)}\right)\times 100,$$

where A indicates the absorbance with enzyme but without sample, B indicates the absorbance without enzyme and sample, C indicates the absorbance with enzyme and sample, and D indicates the absorbance without enzyme but with sample.

### 2.8. Measurement of Elastase Activity

The elastase inhibition test on TCLW and TCLWH was investigated using elastase derived from porcine pancreas. Elastase (500 U) was dissolved in 5 mL of 10 mM tris buffer solution (pH 6.0) and 5 mg elastase substrate IV was dissolved in 5 mL of 100 mM tris buffer solution (pH 8.0). To measure elastase activity, 100 *μ*L of 100 mM tris buffer solution (pH 8.0), 25 *μ*L of elastase substrate IV solution, 50 *μ*L of sample solution, and 25 *μ*L of elastase solution were dispensed into each well of a 96-well plate and then preincubated for 20 min at room temperature. The elastase activity was quantified by measuring light absorbance at 405 nm using a microplate reader (Tecan, Grodig, Austria). Each assay was carried out in triplicate. 

The inhibition rate of elastase was calculated by the following equation:

(3) $$\text{Inhibition}\;\left(\text{\%}\right)=\left(1-\frac{\left(\text{C}-\text{D}\right)}{\left(\text{A}-\text{B}\right)}\right)\times 100,$$

where A indicates the absorbance with enzyme but without sample, B indicates the absorbance without enzyme and sample, C indicates the absorbance with enzyme and sample, and D indicates the absorbance without enzyme but with sample.

### 2.9. Cell Culture

Human foreskin fibroblasts (Hs68) were obtained from neonatal foreskins and cultured in DMEM supplemented with 10% fetal bovine serum, 100 U/mL penicillin, and 100 U/mL streptomycin in a humidified atmosphere of 5%  CO_2_ at 37°C. Hs68 cells were plated at 80%–90% confluence in all experiments.

### 2.10. MTT Assay for Cell Viability

 The fibroblasts were plated at a density of 10^4^ cells/well in a 96-well plate and then treated with various concentrations of extracts dissolved in DMSO (<0.1%) for 24 h. Mitochondrial dehydrogenase activity, which can be used as an index of cell viability, was assessed using the MTT assay as previously described [17]. Viability was quantified by measuring the absorbance at 570 nm using a microplate reader (Tecan, Grodig, Austria).

### 2.11. UV Irradiation

Cells were cultured until 80% confluent, washed twice with phosphate-buffered saline (PBS), and then exposed to UVB irradiation in PBS (302 nm, CL-1000M, UVP, USA). In our previous study, the dose of 80 mJ/cm^2^ UVB irradiation was determined to induce MMP without being cytotoxic (data not shown). Subsequently, cells were incubated for 24 h in 37°C in a humidified atmosphere of 5%  CO_2_ in serum-free DMEM containing various concentrations of TCLW and TCLWH.

### 2.12. Western Blotting Analysis

Western blotting assay was performed using whole cell lysates prepared from Hs68 cells at a density of 5 × 10^5^ cells. Cells were harvested and homogenized with lysis buffer containing 10 mM Na_3_VO_4_, 10 mg/mL leupeptin, 10 mg/mL PMSF and RIPA, and then the lysates were subjected to centrifugation at 12000 ×g for 10 min. All reactions were performed in triplicate. Protein concentration in the culture medium was measured using Bradford reagent (Bio-Rad, Hercules, CA, USA) with bovine serum albumin as the standard. Cell lysates containing equal amounts of total protein were separated by electrophoresis on SDS-polyacrylamide gel and then transferred to a PVDF membrane (Hybond ECL, Amersham Pharmacia Biotech Inc., Piscataway, NJ, USA). Nonspecific binding was blocked with nonfat milk in TBST ((10 mM Tris-HCl, pH 7.5, 150 mM NaCl) containing 0.05% Tween 20). The membrane was incubated with goat polyclonal antibodies against MMP-1 (1 : 500) and type I procollagen (1 : 500), and mouse polyclonal antibodies against MMP-3 (1 : 500), MMP-9 (1 : 500), ERK (1 : 500), JNK (1 : 500), p38 (1 : 500), p-ERK (1 : 500), p-JNK (1 : 500) and p-p38 (1 : 500) (Santa Cruz Biotechnology, Inc., Santa Cruz, CA, USA). Anti-immunoglobulin G-horseradish peroxidase (Santa Cruz Biotechnology Inc.) was used as the secondary antibody. Immunoreactive proteins were detected with the ECL Western blotting detection system (Fujifilm, LAS-4000, Japan). Signal strengths were quantified using a densitometric program (multi Gauge V2.2).

### 2.13. Statistical Analysis

Each experiment was performed in triplicate and all data are presented as mean ± SD. Significant differences between groups were analyzed by ANOVA followed by the Scheffe's test. A *P* value <.05 was considered significant.

## 3. Results

### 3.1. Extraction Yield and Total Phenolic Content of TCLW

The extraction yield of TCLW was 22.5% and that of TCLM was 13.5%. The total phenolic content, expressed as *μ*g gallic acid equivalents (GAE) per mg of dry weight (TCL), was 102.0 ± 0.2 *μ*g GAE/mg.

### 3.2. Scavenging of DPPH Radicals

**Figure 1:**
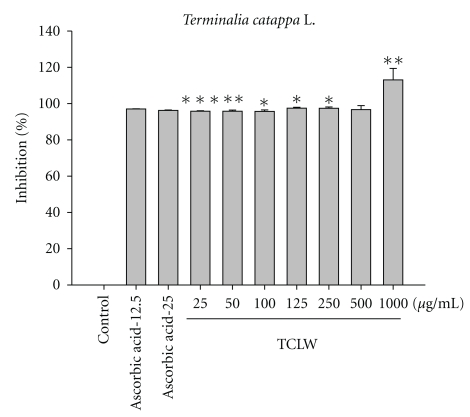
DPPH radical scavenging activity of TCLW. The TCLW provided a strong inhibitory effect and in a dose-dependent manner (25–1000 *μ*g/mL) on DPPH scavenging. (*n* = 4; *P < .05; **P < .01; ***P < .001).

Figure 1 shows the free radical scavenging activity of TCLW (25–1000 *μ*g/mL) and ascorbic acid (25 mg/mL); TCLW exhibited excellent DPPH radical scavenging activity. Our results indicated that DPPH radical scavenging activity of TCLW at 25 *μ*g/mL (95.8 ± 0.3%) was similar to that of ascorbic acid at an equal concentration (96.2 ± 0.2%).

### 3.3. Erythrocyte Hemolysis Assay

**Figure 2:**
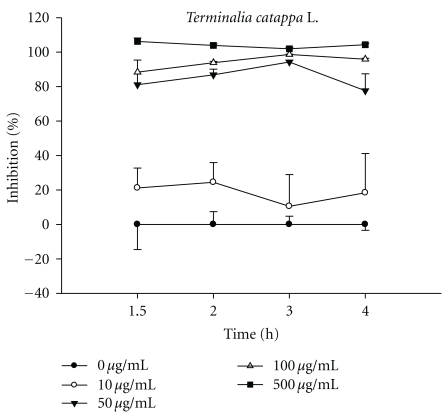
The time course inhibition of TCLW on AAPH-induced lysis of rat erythrocyte. The TCLW provided a strong inhibitory effect and in a dose-dependent manner (50–500 *μ*g/mL) against erythrocyte hemolysis.

The influence of TCLW (10–500 *μ*g/mL) on in vitro erythrocyte hemolysis was examined by incubating rat erythrocytes in the presence of 25 mM AAPH as an initiator of oxidation. TCLW (50–500 *μ*g/mL) exhibited a strong dose-dependent inhibitory effect against erythrocyte hemolysis (Figure 2).

### 3.4. TCLW and TCLWH Inhibited Bacterial Collagenase-1

**Figure 3:**
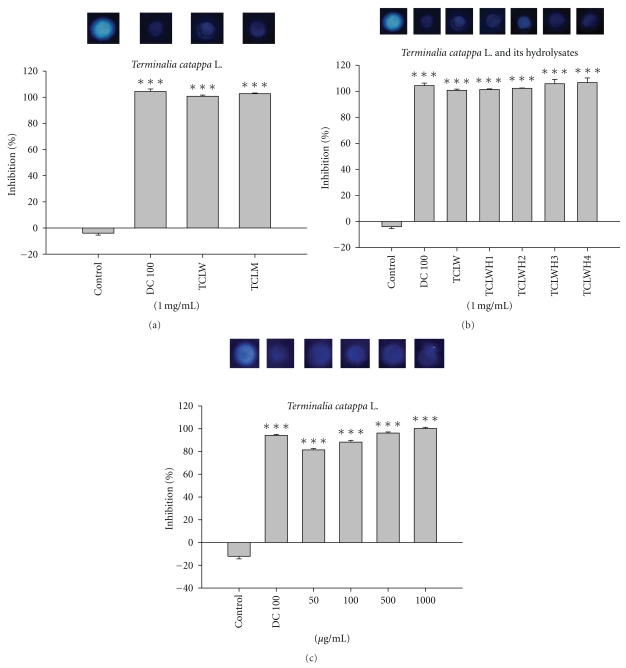
The inhibition of *Terminalia catappa* L. water extract, methanol extract, and its hydrolysates on collagenase activity. (a) *Terminalia catappa* L. water extract (TCLW), and methanol extract (TCLM), (b) *Terminalia catappa *L. water extract (TCLW) and its hydrolysates 1 mg/mL, and (c) *Terminalia catappa *L. water extract (TCLW) 0.05–1 mg/mL (H1: hydrolyzed by 0.6 N HCl for 30 min; H2: 1.2 N HCl for 30 min; H3: hydrolyzed by 0.6 N HCl for 60 min; H4: 1.2 N HCl for 60 min) (positive control, DC 100: doxycycline 100 *μ*g/mL; control: dd water) (*n* = 4; *P < .05; **P < .01; ***P < .001).

For visual investigation of the inhibitory effect of TCLM, TCLW, and TCLWH on MMPs, an indirect assay was developed using bacterial collagenase-1. Following incubation of bacterial collagenase-1 with various concentrations of TCLW and TCLWH, the inhibition of enzyme activity was compared with enzyme activity of the control. As Figure 3(a) shown, DMSO as the control group reaction with the reagent exhibited the highest gelatinolytic activity in the discrete zone; the doxycycline, a well-known MMP inhibitor, was the positive control resulting clear zone. The collagenase-1 inhibition of TCLW and TCLM was similar to doxycycline. As shown in Figure 3(b), the rates of collagenase-1 inhibition were 94.3 ± 0.8% for doxycycline (100 *μ*g/mL), 100.7 ± 0.9% for TCLW (1000 *μ*g/mL), 101.4 ± 0.5% for TCLWH1, 102.2 ± 0.4% for TCLWH2, 105.8 ± 3.4% for TCLWH3, and 106.7 ± 3.5% for TCLWH4. The rates for collagenase-1 inhibition for various concentrations (50–1000 *μ*g/mL) of TCLW ranged from 81.9 ± 0.7% to 100.7 ± 0.7%. In addition, TCLW inhibited gelatinolytic activity in a dose-dependent manner (Figure 3(c)).

### 3.5. TCLW Inhibited Bacterial Collagenase-1

**Figure 4:**
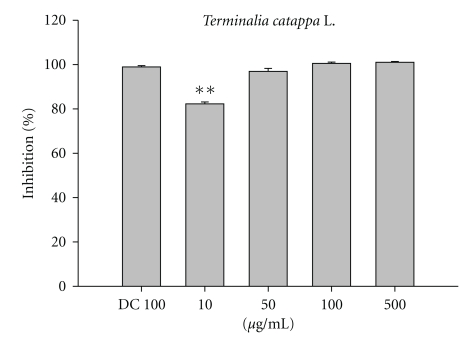
The inhibition rate (%) of TCLW on bacterial collagenase activity using fluorometric assay. (*n* = 4; **P < .01).

Fluorescence-conjugated gelatin was used to measure the inhibitory effect of TCLW on bacterial collagenase-1 protein expression. Fluorescence-conjugated substrate was incubated with bacterial collagenase-1 for 20 h in the presence of different concentrations of TCLW or doxycycline hyclate (positive control) at 37°C. TCLW exhibited a significant inhibitory effect on bacterial collagenase-1; the inhibition rate was >95% of the control at concentrations ≥50 *μ*g/mL (Figure 4).

### 3.6. The Effect of TCLW on Elastase Activity

**Figure 5:**
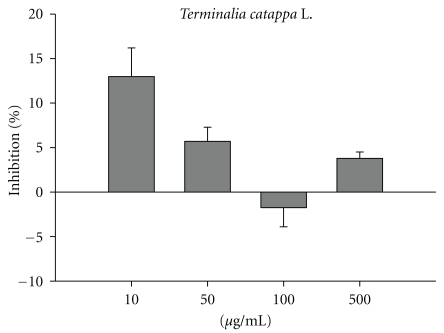
The inhibition rate of TCLW on porcine elastase. (*n* = 4).

This assay measures the synthesis and activity of elastase in cells exposed to TCLW. As shown in Figure 5, TCLW (10–500 *μ*g/mL) did not have a significant effect on elastase activity.

### 3.7. TCLW Inhibited the Protein Expression of MMPs

**Figure 6:**
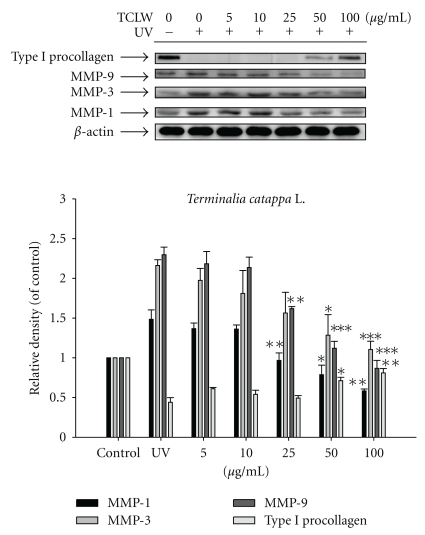
Effect of TCLW on the UVB-induced expression of MMP-1, 3, 9, and type I procollagen in human fibroblasts. (*n* = 4; *P < .05; **P < .01; ***P < .001).

UVB irradiation of untreated Hs68 cells resulted in a 1.5-fold increase in MMP-1 expression, a 2.2-fold increase in MMP-3 expression, and a 2.3-fold increase in MMP-9 expression relative to control levels. TCLW treatment (25–100 *μ*g/mL), however, suppressed the UVB-induced upregulation of MMPs (Figure 6). TCLW at concentrations of 25 *μ*g/mL and higher led to a significant decrease in MMP-1 and -9 expression to basal level. TCLW treatment at concentrations of 50 *μ*g/mL and higher significantly inhibited MMP-3 expression (Figure 6).

### 3.8. TCLW Upregulates Type I Procollagen Expression

Fibroblasts were treated with TCLW (5–100 *μ*g/mL) for 24 h after exposure to UVB (80 mJ/cm^2^). TCLW treatment (≥50 *μ*g/mL) led to a significant increase in the expression of type I procollagen (Figure 6).

### 3.9. Effect of TCLW on MAP Kinase Expression

**Figure 7:**
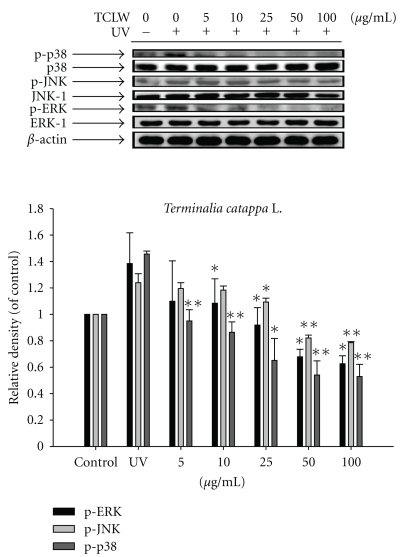
Effect of TCLW on the UVB-induced expression of MAP kinases in human fibroblasts. (*n* = 4; *P < .05; **P < .01).

UVB irradiation of untreated Hs68 cells resulted in a 1.4-fold increase in p-ERK expression, a 1.2-fold increase in p-JNK expression, and a 1.5-fold increase in phosphorylated p38 relative to control levels. TCLW treatment (5–100 *μ*g/mL), however, significantly inhibited the UVB-induced overexpression of those MAP kinases. P-ERK was suppressed to the basal level at 10 *μ*g/mL TCLW, p-JNK was suppressed to the basal level at 25 *μ*g/mL TCLW, and phosphorylated p38 was suppressed to the basal level at 5 *μ*g/mL TCLW (Figure 7).

### 3.10. Effect of TCLW on Cell Viability

**Figure 8:**
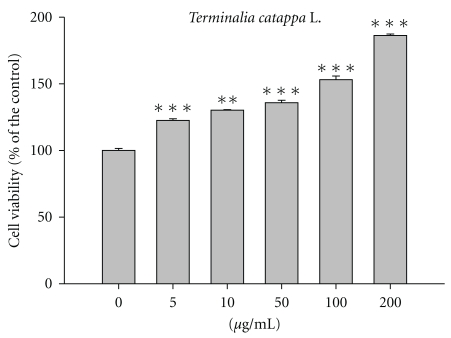
Cell viability of TCLW on human fibroblasts. (*n* = 4; **P < .01; ***P < .001).

Hs68 cells were treated with various concentrations of TCLW (5–200 *μ*g/mL) and cell viability was measured. As shown in Figure 8, TCLW did not exhibit cytotoxic effects on the proliferation of cells (cell viability >90% of control). In addition, TCLW promoted cell proliferation in a dose-dependent manner.

## 4. Discussion

Aging is a complex phenomenon that is modulated by multiple factors, including genetics, life style, and exposure to sunlight and pollutants. UV radiation produces ROS, which activate MAPkinase pathway to induce the expression and activation of MMPs that degrade extracellular matrix proteins including type I collagen, elastin, and glycosaminoglycans in skin [18, 19].

It has been shown that *Moringa oleifera *extract, which has a total phenolic content of 52.5 *μ*g GAE/mg, has potent DPPH scavenging activity [20]. In addition, *Fraxinus chinensis *extract scavenges UVA-induced DPPH free radicals and inhibits MMP-1 mRNA and protein expression in human skin fibroblasts [21]. * Sorbus commixta* Hedl, a compound with a high total phenolic content, exhibits free radical scavenging effect and decreases MMP-1 mRNA expression [22]. *Terminalia catappa *L. leaves are rich in polyphenol antioxidants. In addition, studies have shown that the antioxidant activities of polyphenols reduce the risk of skin diseases [23]. We speculate that TCLW can protect against oxidative stress-induced photodamage because of its high total phenolic content (102 *μ*g GAE/mg), DPPH free radical scavenging activity, as well as its ability to prevent AAPH-induced hemolysis and activate MMP.

UV stimulates collagenase expression, which causes matrix protein degradation and, subsequently, skin photoaging [24, 25]. Collagenase-1 (MMP-1) degrades collagen, gelatin, and proteoglycan link protein, and collagenase-3 (MMP-3) is involved in several MMP activation cascades including activation of MMP-1 [26]. Agents that inhibit collagenase activity would be ideal candidates for the prevention or treatment of photoaging. Botanical extracts have been reported to suppress collagenase activity. For example, *Viscum coloratum* inhibits collagenase release [27] and *Morinda citrifolia* inhibits elastase and tyrosinase activities [28]. In addition, xanthorrhizol, a sesquiterpenoid isolated from ethyl acetate extract of *Curcuma xanthorrhiza, *inhibits UVB-induced MMP-1 expression and increases the level of type I procollagen in human fibroblasts [29]. Erythrodiol-3-acetate from *Spiraea japonica *stem extract has been shown to upregulate type I procollagen expression in fibroblasts after exposure to UVB irradiation [30]. Furthermore, *Magnolia obovata* extract (150 *μ*g/mL) and *Polypodium leucotomos* (50 *μ*g/mL) have been reported to attenuate MMP-1 expression [31, 32]. We found that TCLW (25 *μ*g/mL) inhibited MMP-1, -3 and -9 expression, a finding consistent with that reported by Yang et al. [33].

**Figure 9:**
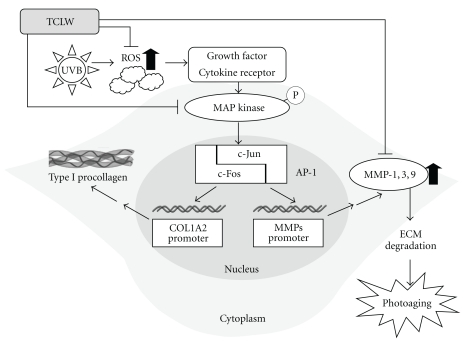
The mechanisms of TCLW on UVB-induced photodamage.

MAP kinases are upstream regulators of MMP activity [34–36]. It has been reported that green tea polyphenol, epigallocatechin-3-gallate (EGCG) inhibits the expression of MMPs by attenuating the phosphorylation of ASK-1 and MAPkinase pathway [37]. In addition, treatment of UV-irradiated cells with 4-hydroxypanduratin A, isolated from *Kaempferia pandurata* Roxb, leads to decreased MMP-1 expression by inhibiting c-Fos and c-Jun phosphorylation and MAP kinase pathway activation [38]. In this study, TCLW significantly inhibited UVB-induced p-ERK, p-JNK, and phosphorylated p38 expression at doses of 10, 25, and 5 *μ*g/mL, respectively. It has been shown that UV irradiation induces p38 activation [34]. Furthermore, it has been reported that UVB irradiation induces ERK, JNK, and p38 activation via production of ROS [39, 40]. Green tea polyphenol, EGCG, suppresses UV-induced oxygen stress-mediated MAP kinase phosphorylation pathway [41]. The flavonoid, fisetin inhibits UV-induced MAP kinase expression and NF-*κ*B signal transduction [42, 43]. In our study, TCLW exhibited potent antioxidant activity, inhibited the expression of MAP kinases and MMPs, and activated type I procollagen. Those findings indicate that TCLW is a potential agent for the treatment or prevention of photodamage. TCLW hampered the activation of MAPKs, and, therefore, may downregulate the down stream proteins c-Fos and c-Jun, as well as the transcription factor AP-1 and protooncogenes involved in photodamage (Figure 9). The effects of TCLW on transcription of c-Fos, c-Jun, and AP-1 need further study.

## 5. Conclusions

TCLW inhibits UVB-induced ROS, phosphorylation of p38, JNK, and ERK, and attenuates the expression of MMP-1, -3, -9, thereby elevating type I procollagen synthesis. TCLW, therefore, could be a potential antiphotodamage agent.
